# Prevalence of depressive symptoms and suicide risk among medical residents

**DOI:** 10.1192/j.eurpsy.2022.1412

**Published:** 2022-09-01

**Authors:** C. Reyes, V. Santana, G. Arocha, N. Martínez, K. Almonte

**Affiliations:** Pontificia Universidad Católica Madre y Maestra, Escuela De Medicina, Santiago De Los Caballeros, Dominican Republic

**Keywords:** Suicide, Depression, mental health, medical residents

## Abstract

**Introduction:**

Depression and suicide risk are disturbing issues within the medical community. In many countries, physician’s mental health is not a concern, due to the fact that many do not even consider medical staff as potential mental health patients. However, health care providers are an at risk population for phycological affliction due to their heavy workload.

**Objectives:**

We aim to describe the prevalence of depressive symptoms and suicidal risk among medical residents from health centers of Santiago de los Caballeros, Dominican Republic.

**Methods:**

A cross-sectional descriptive study was made, between the months of February and May 2021, using the Beck Depression Inventory II (BDI-2) and the Plutchick Suicidal Risk Scale.

**Results:**

There was a total population of 507 residents, where 231 completed the survey. Of these, 1 recanted his participation, and 14 were excluded according to the study’s criteria, resultingin a total of 217 residents. The overall prevalence of depressive symptoms was 24.9% and suicidal risk was 22.94%. Residents who worked in a private center had 3.83 times more risk of suffering depressive symptoms compared to those who belonged to the public sector. Furthermore, residents from Internal Medicine (39.5%) had a higher prevalence of depressive symptoms, and residents from Anesthesiology (42.2%) suffered a higher suicide risk compared to other medical residences.

**Conclusions:**

A disturbing percentage of the medical residents suffer from depressive symptoms and suicidal risk. Therefore, residency programs should offer assistance to help prevent and manage mental health disorders.

**Disclosure:**

No significant relationships.

